# Comparative analysis of humoral immune responses and pathologies of BALB/c and C57BL/6 wildtype mice experimentally infected with a highly virulent *Rodentibacter pneumotropicus* (*Pasteurella pneumotropica*) strain

**DOI:** 10.1186/s12866-018-1186-8

**Published:** 2018-05-30

**Authors:** Juliane Fornefett, Jaqueline Krause, Kristin Klose, Felix Fingas, Rayk Hassert, Laurentiu Benga, Thomas Grunwald, Uwe Müller, Wieland Schrödl, Christoph Georg Baums

**Affiliations:** 1 Institute for Bacteriology and Mycology, Faculty of Veterinary Medicine, University Leipzig, An den Tierkliniken 29, 04103 Leipzig, Germany; 2 Fraunhofer Institute for Cell Therapy and Immunology, Leipzig, Germany; 3 Institute for Veterinary Pathology, Faculty of Veterinary Medicine, University Leipzig, Leipzig, Germany; 4 GVG Diagnostics GmbH, Leipzig, Germany; 5 Institute of Bioanalytical Chemistry, Faculty of Chemistry and Mineralogy and Centre for Biotechnology and Biomedicine, University Leipzig, Leipzig, Germany; 6 Central Unit for Animal Research and Animal Welfare Affairs, Heinrich-Heine-University, University Hospital, Düsseldorf, Germany; 7 Institute of Immunology/Molecular Pathogenesis, Faculty of Veterinary Medicine and Centre for Biotechnology and Biomedicine, University Leipzig, Leipzig, Germany

**Keywords:** Bronchopneumonia, RTX toxins, Th1/Th2 responses, Colonization, Animal model

## Abstract

**Background:**

Mice are a natural host for *Rodentibacter (R.) pneumotropicus.* Despite specific monitoring, it is still one of the most important infectious agents in laboratory animals. The objective of this study was to determine the virulence of a prevalent pathotype of *R. pneumotropicus* and characterize the host response in a new animal model.

**Results:**

Intranasal infection of C57BL/6 and BALB/c mice with a *R. pneumotropicus* strain (JF4Ni) bearing the genes of the three known repeats in toxin (RTX) toxins resulted in an unprecedented high mortality and morbidity above 50 and 80%, respectively. Morbidity was associated with severe weight loss as well as conjunctivitis and dyspnea. A main pathology was a catarrhal purulent to necrotic bronchopneumonia. Specific immune globuline (Ig) A was detected in tracheonasal lavages of most surviving mice which were still colonized by *R. pneumotropicus*. Furthermore, all surviving animals showed a distinct production of IgG antibodies. To differentiate T-helper cell (Th) 1 and Th2 immune responses we used subclasses of IgGs as indicators. Mean ratios of IgG2b to IgG1 were below 0.8 in sera drawn from both mice strains prior infection and from BALB/c mice post infection. In contrast, C57BL/6 mice had a mean IgG2b/IgG1 ratio of 1.6 post infection indicating a Th1 immune response in C57BL/6 versus a Th2 response in BALB/c mice associated with a tenfold higher bacterial load in the lung. In accordance with a Th1 response high antigen-specific IgG2c titers were detected in the majority of surviving C57BL/6 mice.

**Conclusions:**

*R. pneumotropicus* JF4Ni is a highly virulent strain causing severe pneumonia and septicemia after intranasal infection of C57BL/6 and BALB/c mice. Persisting infections in the two mice strains are associated with Th1 and Th2 immune responses, respectively, and differences in the bacterial burden of the lung. The described model is ideally suited for future vaccination studies using the natural host.

**Electronic supplementary material:**

The online version of this article (10.1186/s12866-018-1186-8) contains supplementary material, which is available to authorized users.

## Background

*Pasteurella (P.) pneumotropica* was thought to be a species occurring mainly in two different biotypes: *Jawetz* and *Heyl* [1, 2]. However, *P. pneumotropica* was very recently reclassified and these two biotypes belong now to two different species, namely *Rodentibacter* (*R.*) *pneumotropicus* and *R. heylii*, respectively [3]. The differentiation of the two biotypes is based on the phenotype of the colony colour (grey and yellow, respectively), but polymerase chain reaction (PCR)-based differentiation is also possible [4–6]. In a recent study [7] differences in distribution of virulence factors between the two biotypes are described. Three different repeats-in-toxin (RTX) toxins, designated PnxI, PnxII and PnxIII, have been identified in *P. pneumotropica*. Whilst PnxI and PnxII are secreted and act as haemolysins or cytotoxins [8], PnxIII is associated with the bacterial membrane. Specifically, PnxIII interacts with the extracellular matrix [9, 10] but can also induce host cell cytotoxicity [9, 10]. Therefore, these RTX toxins are considered as important virulence factors [9].

*P. pneumotropica* is among the most important pathogens in laboratory animal populations with a reported prevalence of 4 to 13% in Europe and North America, respectively [11]. It is described as an opportunistic pathogen with low virulence in immunocompetent mice [2] but clinical signs were recorded in immunodeficient and –suppressed mice or in co-infections with *Mycoplasma pulmonis* [12] or *Pneumocystis carinii* [13]. Infected animals are generally unsuitable for scientific research due to suppurative to necrotizing lesions in various organs [14] and modulation of the immune response [15]. The Federation of Laboratory Animal Science Association (FELASA) lists *P. pneumotropica* as an important pathogen in mice, rats and hamsters and recommends the examination every 3 months [16]. Various monitoring methods are described including PCR [4, 5, 17] and indirect enzyme-linked immunosorbent assays (ELISAs) [18–20].

It is common practice to use soiled bedding sentinels for health monitoring of laboratory animals. Nevertheless, studies revealed a limited survival of *P. pneumotropica* in the environment [21, 22] and the failure of detecting *P. pneumotropica* infections by bedding sentinels [23].

In this study, we evaluated the pathologies and immune responses induced by experimental infection of BALB/c and C57BL/6 mice with a *R. pneumotropicus* pathotype emerging in German laboratory animal facilities. The new intranasal model leading to severe pneumonia, septicaemia but also to persisting infections is important for future studies on virulence and protection.

## Results

### Distribution of *pnx*IA*, pnx*IIA and *pnx*IIIA in *R. pneumotropicus* and *R. heylii*

Different genes encoding RTX-toxins have been identified in *P. pneumotropica,* namely *pnx*IA*, pnx*IIA and *pnx*IIIA. By PCRs targeting these *pnx* genes, we investigated recently collected 27 *R. pneumotropicus* and 26 *R. heylii* strains. This profiling revealed that the distribution of *pnx*IA*, pnx*IIA and *pnx*IIIA differs substantially between the two species (Table 1). In 46% of the *R. heylii* strains only *pnx*I was detected. The gene *pnx*II was not found at all in this species. Twelve percent of the *R. heylii* strains carried both *pnx*III and *pnx*I, whereas 43% were PCR negative for all RTX genes. In contrast, all three RTX genes were found in 74% of the *R. pneumotropicus* strains. Only 7% of the *R. pneumotropicus* strains did not carry any of the *pnx* genes. For further investigation, a recently isolated *pnx*IA*+, pnx*IIA*+* and *pnx*IIIA*+ R. pneumotropicus* strain (JF4Ni) was chosen, because of its prevalent genotype identified by the screening of strains collected in Germany.

**Table 1:** Distribution of RTX genes *pnx*IA, *pnx*IIA and *pnx*IIIA in *R. pneumotropicus and R. heylii*

	only *pnx*IA	only *pnx*IIA	only *pnx*IIIA	*pnx*IA + IIA	*pnx*IA + IIIA	*pnx*IIA + IIIA	*pnx*IA + IIA + IIIa	none
*R. pneumotropicus* (*n* = 27)	7%	0%	0%	4%	4%	4%	74%	7%
*R. heylii* (*n* = 26)	46%	0%	0%	0%	12%	0%	0%	42%
in total (*n* = 53)	26%	0%	0%	2%	8%	2%	38%	24%

### Morbidity, mortality and histopathology of experimentally infected animals and sentinels

**Fig. 1:**
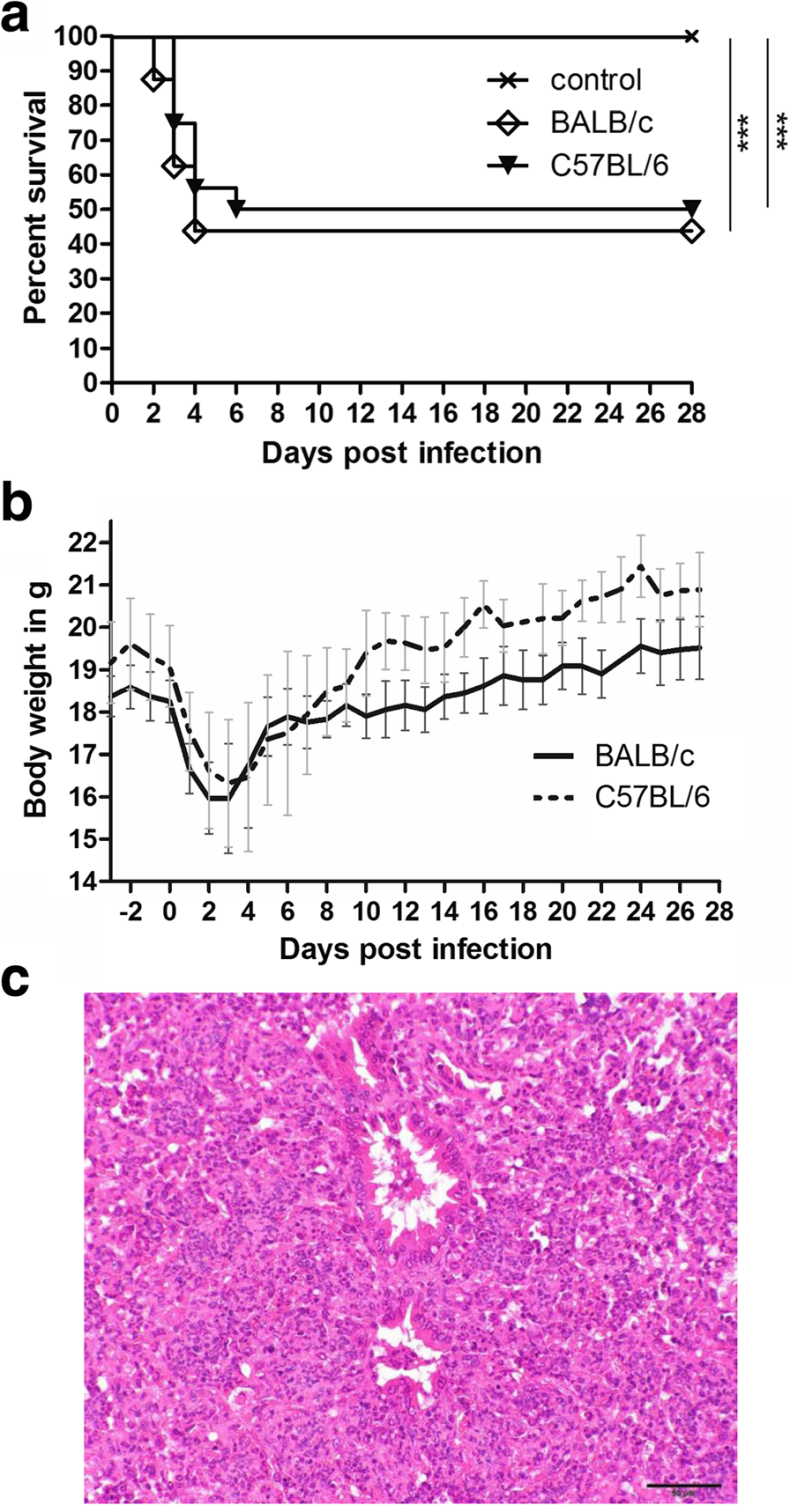
Mortality (**a**) and body weight (**b**) of the indicated mice infected with *R. pneumotropicus* JF4Ni (*n* = 16 per mouse strain) or treated with PBS as control (*n* = 10 for BALB/c and *n* = 9 for C57BL/6, one control died during anaesthesia). Data of contact sentinels are not included. Bronchopneumonia was a main pathology. A multifocal severe catarrhal-purulent bronchopneumonia of a BALB/c mouse 2 days after intranasal infection is shown (**c**). Alveoli and bronchioles of this mouse were infiltrated with high numbers of neutrophilic granulocytes (200 x magnification). The log rank test was used to analyse differences between the two mice strains and the groups (**a**)

Intranasal infection with 10^8^ CFU *R. pneumotropicus* JF4Ni resulted in 100% (16/16) morbidity in BALB/c and 87.5% (14/16) morbidity in C57BL/6 mice within one day. Fifty-six percent (9/16) of BALB/c died or had to be euthanized within 2–4 days after infection and 50% (8/16) of C57BL/6 within 3–6 days after infection (Fig. 1a). Diseased mice showed unspecific signs such as ruffled coat, bended back, heavy weight loss (Fig. 1b), dehydration as well as specific signs such as dyspnoea, conjunctivitis and mild incoordination in two cases. Significant differences in mortality and morbidity were only recorded between infection animals and controls, but not between the two mouse strains. All early deceased mice showed multifocal moderate to severe catarrhal-purulent bronchopneumonia, in some cases with necrosis (Table 2 and Fig. 1c). These pathologies were not recorded in mice which survived the experimental *R. pneumotropicus* infection. A mild to moderate interstitial pneumonia was found in all mice including controls and sentinels. Contact and bedding sentinels showed no clinical signs and pathologies related to *R. pneumotropicus* infection (data not shown).

**Table 2:** Degree and extent of catarrhal-purulent bronchopneumonia in *R. pneumotropicus* infected mice (for definition of scores see Additional file 5: Table S5)

	BALB/c			C57BL/6		
	0	1–3	4–7	0	1–3	4–7
Controls	10/10	0/10	0/10	9/9	0/9	0/9
Losses	0/9	1/9	8/9	1/8	0/8	7/8
Survivors	7/7	0/7	0/7	8/8	0/8	0/8
Contact sentinels	4/4	0/4	0/4	4/4	0/4	0/4

### Detection of *R. pneumotropicus* in tracheonasal lavages (TNL) and internal organs

**Fig. 2:**
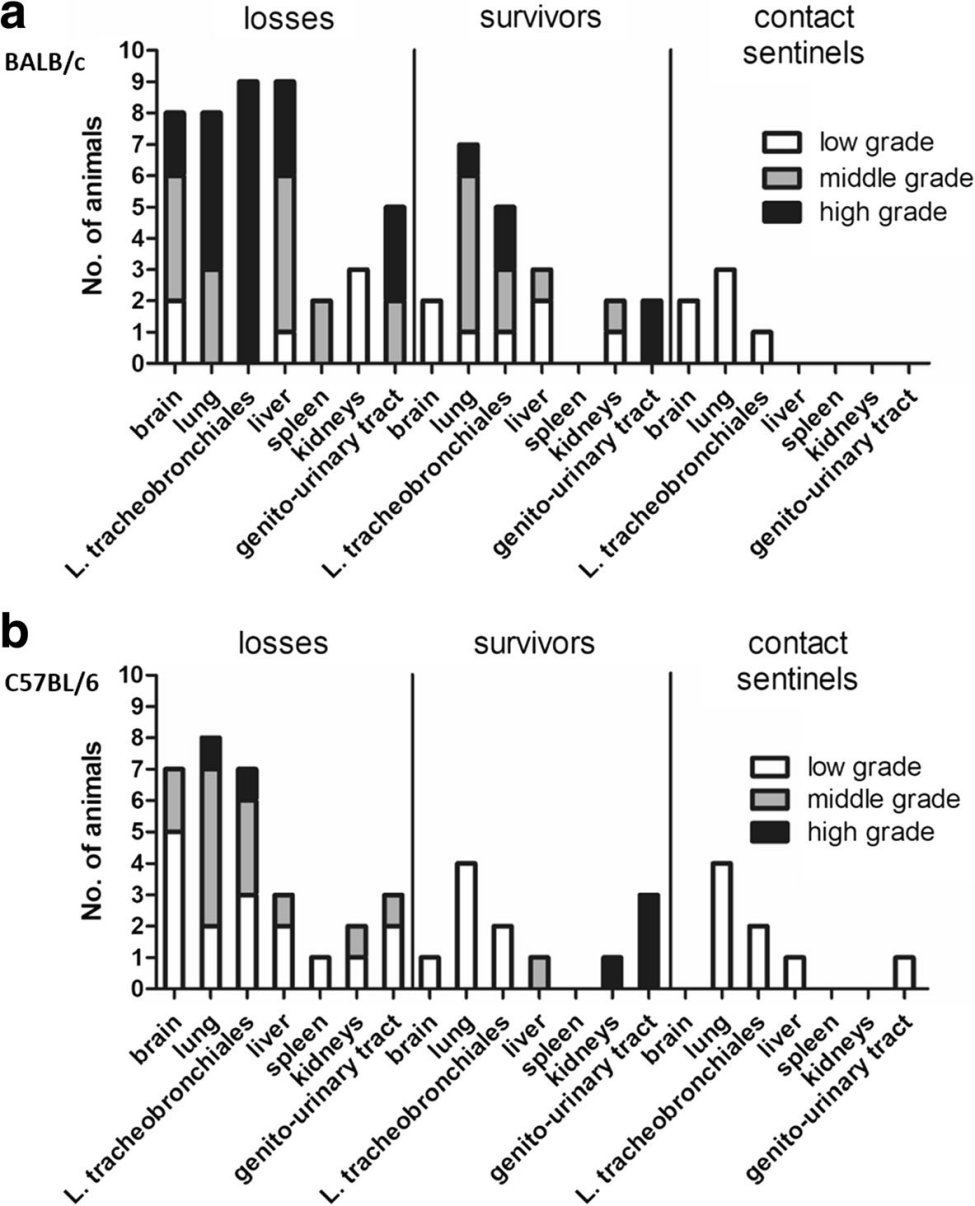
Semi-quantitative determination of *R. pneumotropicus* in the indicated tissues of BALB/c (**a**) and C57BL/6 (**b**) mice either infected experimentally (losses and survivors) or used as contact sentinels. Losses occured 2–6 dpi, survivors were sampled 28 dpi and contact sentinels 56 dpi. A low grade is equal to less than 20 CFU per plate; a middle grade refers to 20–70 CFU and a high grade to more than 70 CFU per plate

*R. pneumotropicus* was detected in TNL and various internal organs of experimentally infected animals. Furthermore, this pathogen disseminated into non-respiratory internal organs in every experimentally infected mouse (Fig. 2). Bacterial loads in the brains, lungs, L n. tracheobronchiales, livers, spleens, kidneys and genito-urinary tracts were significantly higher in BALB/c than in C57BL/6 mice as assessed by semi-quantitative scoring (mean bacteriological scores for mice succumbing to infection: BALB/c: 11.6 with SD 3.0 and C57BL/6: 6.1 with SD 1.9; *p* = 0.002; Additional file 1: Table S1; Fig. 2). In contact sentinels *R. pneumotropicus* was mainly detected in the lungs and TNLs (Fig. 2), indicating that dissemination occurred mainly in experimentally infected animals but not in contact sentinels. Additionally, *R. pneumotropicus* was not detected in bedding sentinels at all.

**Fig. 3:**
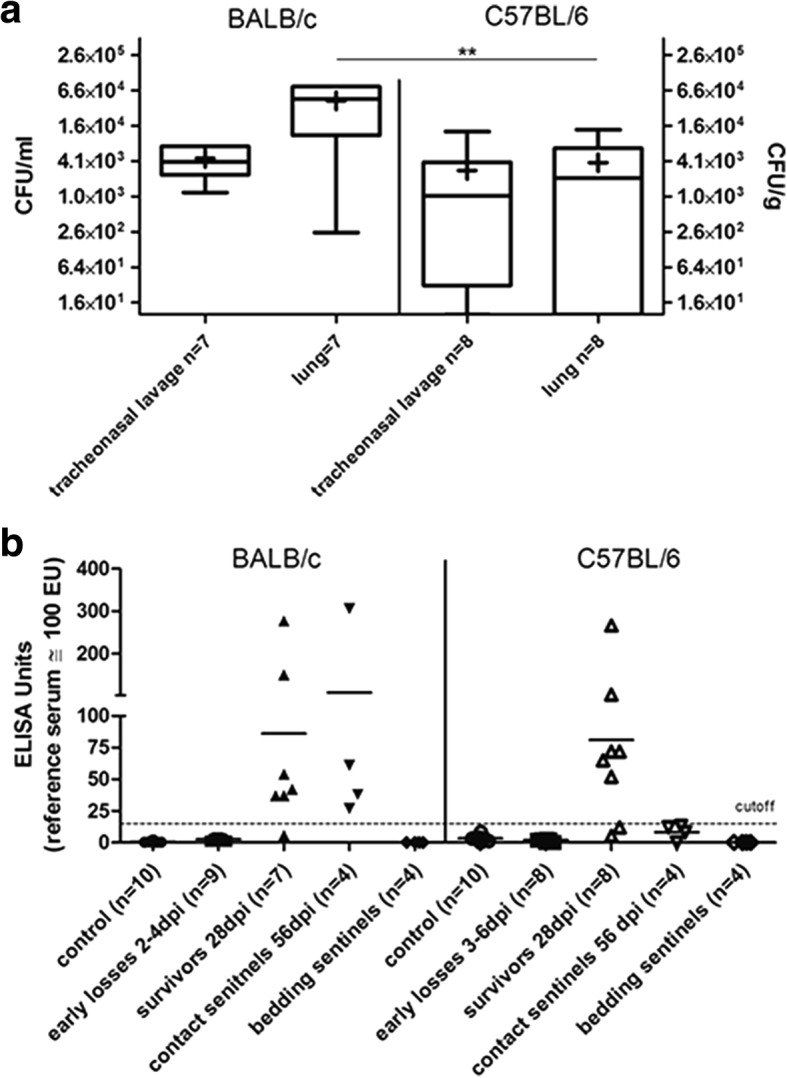
Quantitative determination of *R. pneumotropicus* in TNL and lungs of the indicated mice surviving experimental infection (**a**) and pathogen-specific IgA-levels in TNL (**b**). Medians are marked by the horizontal line. The non-parametric Mann-Whitney test was used for statistical analysis (** for *P* ≤ 0.01)

BALB/c and C57BL/6 mice succumbing to infection within 2 to 6 days post infection (dpi) had high specific bacterial loads in TNL in most cases (mean of 2.6 × 10^6^ colony forming units (CFU)/ml with SD 3.1 × 10^6^ and 3.4 × 10^5^ CFU/ml with SD 3.7 × 10^5^, respectively). At the end of the observation period, the bacterial load of the lung was significantly higher in surviving BALB/c mice than in C57BL/6 mice (mean of 4.4 × 10^4^ CFU per g tissue with SD 3.2 × 10^4^ and mean of 3.9 × 10^3^ CFU per g tissue with SD 5.0 × 10^3^, respectively, Fig. 3a). In contact sentinels, mean *R. pneumotropicus* loads in TNL of 2.8 × 10^4^ CFU per ml TNL in BALB/c and 2.6 × 10^4^ CFU per ml TNL in C57BL/6 were recorded (SD 3.9 × 10^4^ and 1.9 × 10^4^, respectively). Noteworthy, the mean bacteriological score based on semi-quantitative assessment of bacterial loads in the brains, lungs, lymphonodi tracheobronchiales, livers, spleens, kidneys and genito-urinary tracts was significantly higher in surviving BALB/c than in surviving C57BL/6 mice (mean bacteriological scores of 5.7 (SD 2.7) and 2.4 (SD 3.4) in BALB/c and C57BL/6 mice, respectively; *p* = 0.036; Additional file 2: Table S2; Fig. 2).

### Antigen-specific IgA in TNL of experimentally infected animals and sentinels

*As R. pneumotropicus* was isolated in TNL of experimentally infected mice surviving to the end of the observation period, we asked if this colonization occurred in the presence of specific IgA. *R. pneumotropicus*-specific IgA was not detected in TNL of controls and mice that died within 6 days following experimental infection. On the other hand, TNLs collected 4 weeks post infection from experimentally infected mice of both strains and BALB/c contact sentinels revealed mean titers above 80 ELISA units and mainly positive IgA-titers against *R. pneumotropicus* (Fig. 3b). In contrast, C57BL/6 contact sentinels had rather low specific IgA titers (mean titer of 8 ELISA units with a SD of 5.9). In summary *P. pneumotropicus* was found to colonize efficiently respiratory mucosa despite the presence of specific IgA in mice surviving experimental infection.

### Serum IgG-levels in experimentally infected animals and sentinels

**Fig. 4:**
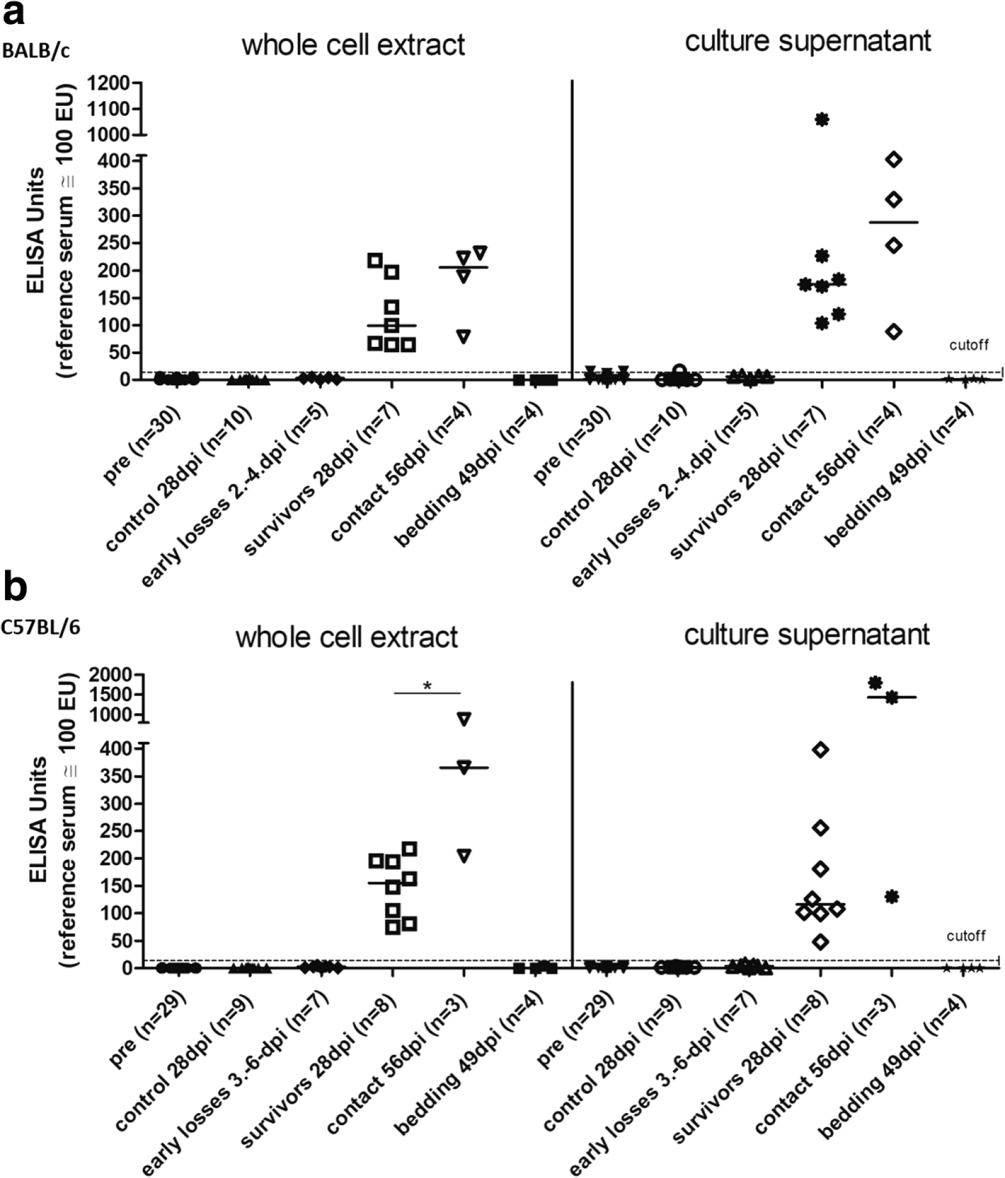
IgG-levels against the indicated *R. pneumotropicus* antigens in intranasally infected BALB/c (**a**) and C57BL/6 (**b**) mice and the respective contact and bedding sentinels in sera drawn at the indicated time points. A serum pool from mice naturally infected with *R. pneumotropicus* was used as reference serum. The culture supernatant had a concentration factor of 100. Early losses refer to mice killed for animal welfare reasons after developing severe signs of sepsis. Statistical analysis with the Mann-Whitney test was performed to analyse differences between the different groups. The Wilcoxon test was used to compare different time point values within the same groups. The star in panel (**b**) (whole cell extract) indicates significance (* for *P* ≤ 0.05)

Experimental infection of BALB/c and C57BL/6 mice elicited specific IgG-titers in all surviving animals (sampled 28 dpi) as well as in contact sentinels (sampled 56 dpi) as shown by ELISA using whole cell extract or a concentrated culture supernatant as antigen (Fig. 4a and b). Sera collected prior to infection (*n* = 59) and from control animals (*n* = 19) consistently gave negative results. Furthermore, *R. pneumotropicus*-specific IgG was not recorded in sera from mice succumbing to infection within the first 6 dpi or in sera from bedding sentinels.

### IgG subclass differentiation indicate differences in the immune response of BALB/c and C57BL/6 mice to *R. pneumotropicus* infection

**Fig. 5:**
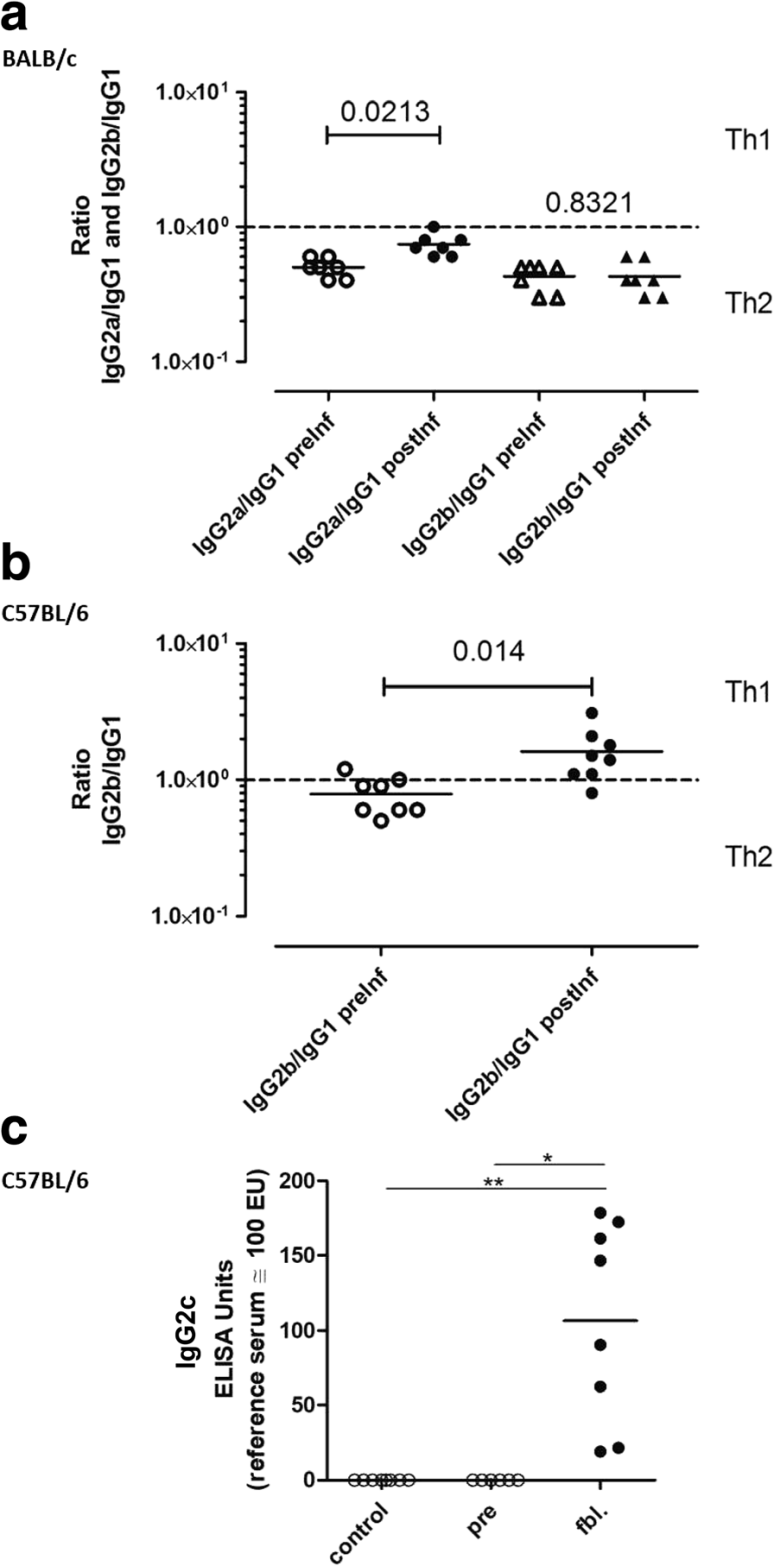
Ratios of the indicated IgG-subclasses in surviving C57BL/6 (**a**) and BALB/c (**b**) mice and antigen-specific IgG2c titers in respective C57BL/6 (**c**). Seven infected BALB/c and 8 C57BL/6 mice with a distinct antibody answer to *R. pneumotropicus* antigen (Fig. 4) were chosen for the determination of IgG subclasses in (**a**) and (**b**), respectively. In BALB/c mice the ratios between IgG2a to IgG1 and IgG2b to IgG1 were determined. As C57BL/6 mice lack IgG2a, only IgG1 and IgG2b were measured. Medians are marked by the horizontal line. Differentiation of a Th1 and Th2 immune response is indicated by the dashed line. IgG2c-titers in C57BL/6 mice against whole cell extract of *R. pneumotropicus* (**c**). Titers were determined as indicated either in experimentally infected survivors or respective controls (fbl = final bleeding). A serum pool from C57BL/6 mice naturally infected with *R. pneumotropicus* was used as reference serum. The non-parametric Mann-Whitney test was used for statistical analysis. The *P*-values are indicated

As the specific bacterial load of the lung of surviving BALB/c mice was significantly higher than the load of C57BL/6 mice (Fig. 3a), we investigated putative differences in the immune response of the two mice strains. We used levels of IgG subclasses as indicators for the kind of immune response as IgG2a and IgG2b are associated with a Th1 response while IgG1 is associated with a Th2 response. Upon infection, not only the ratio of antigen-specific IgG subtypes might change in serum in association with a Th1 or Th2 response, but also the overall ratios of IgG subtypes [24]. Differentiation of IgG subclasses was conducted in 7 BALB/c and 8 C57BL/6 mice showing a distinct antibody response to *R. pneumotropicus* antigen. In BALB/c mice, we measured the ratios of IgG2a to IgG1 and IgG2b to IgG1. Due to the lack of IgG2a in C57BL/6 mice [25] only the ratio of IgG1 to IgG2 in was determined in this strain. Prior to infection, the mean IgG2/IgG1 ratios were 0.5 for IgG2a and 0.4 for IgG2b in BALB/c and 0.8 for IgG2b in C57BL/6 mice (Fig. 5a and b). Post infection, the mean IgG2a/IgG1 ratio in BALB/c mice increased slightly to 0.7, while the IgG2b/IgG1 ratio remained constant. These results indicate a balanced immune response with a tendency to Th2 in BALB/c mice. In C57BL/6 the IgG2b to IgG1 ratio increased to 1.6 indicating a Th1 prone immune response (Fig. 5a). Accordingly, the majority of surviving, experimentally infected C57BL/6 mice had high *R. pneumotropicus*-specific IgG2c titers (above 50 ELISA Units), whereas these antibodies were not recorded in sera drawn from these mice prior infection or from control mice (Fig. 5c).

## Discussion

Experimental infections with *P. pneumotropica* have been conducted in vaccination studies to investigate the protective efficacies of different recombinant proteins such as the RTX toxin PnxIII [9] and different outer membrane proteins [26]. In these studies, protective efficacies were assessed by determining specific bacterial loads at different mucosal sites such as the nasal conchae, lung and conjunctivae. However, clinical read out parameters were not included. Here, an infection model with high rates of morbidity and mortality in immunocompetent BALB/c and C57BL/6 wildtype mice was established for the first time, enabling the usages of respective read out parameters in future vaccination studies. The high morbidity was associated with severe lung pathologies and dissemination of *R. pneumotropicus* to extra-respiratory sites such as liver and brain. Based on the presented data we consider these models as ideal for investigating protection against severe pneumonia and associated sepsis. Importantly, numerous immunogens of *R. pneumotropicus* are homologous to proteins expressed by *Haemophilus influenza* [26] and *P. multocida* [26], two important pneumonia and sepsis agents in humans and livestock, respectively. Thus, vaccination trials using the described murine models might be relevant for these pathogens as well. The fact that mice are the natural host of *R. pneumotropicus* is an important advantage, especially as members of the Pasteurellaceae show substantial host adaptation.

The infection dose of 1 × 10^8^ CFU used in this study is high. However, previous studies have used similar or even higher [15, 27, 28] doses without induction of morbidity and mortality. For example, Chapes et al. could not induce any clinical signs or pathologies in immunocompetent C57BL/6 wildtype mice with a 400 times higher dose of the *R. pneumotropicus* type strain ATCC 35149 [27]. Furthermore, immunocompetent Crlj:CD1 mice did not develop lung lesions or clinical signs of disease after experimental infection with the *R. pneumotropicus* type strain in contrast to immunodeficient NOD/ShiJic-scid/Jcl mice [28]. This difference to previous studies might be related to differences in virulence between *R. pneumotropicus* strains. The *R. pneumotropicus* JF4Ni strain used in this study is regarded as highly virulent based on the results of the experimental infections and the substantial health issues of the animal facility it was detected in. The fact that this strain bears all three known RTX toxin genes of this pathogen might be related to its virulent phenotype as the toxins are considered important virulence factors [8–10]. Specifically, PnxIII is cytotoxic to macrophages [10], which are crucial for clearance of *R. pneumotropicus* in the lungs as shown by transfer of Toll-like receptor 4-positive macrophages to knockout mice [29]. Noteworthy, more than 70% of the *R. pneumotropicus* strains investigated in this study shared this RTX toxin genotype, namely *pnx*IA+ *pnx*IIA+ *pnx*IIIA+. As these strains were recently isolated, putative emergence of this important pathotype should be further investigated.

All surviving animals of both mouse strains and their contact sentinels produced high levels of *R. pneumotropicus*-specific IgG antibodies, as shown by ELISA using two different antigens. Sera drawn prior to infection and from control animals consistently gave negative results which indicates high specificities of the established ELISAs. Noteworthy, a commercially available ELISA for the detection of *P. pneumotropica*-specific IgG failed to detect specific antibodies in the sera of infected animals surviving until the end of the experiment (results not shown).

In agreement to previous studies, all bedding sentinels were serologically negative, rendering this approach inappropriate for *R. pneumotropicus* health monitoring [21, 23]*.* On the other hand, serological screenings of contact sentinels for *R. pneumotropicus*-specific serum IgG using the described ELISA seems to be rather sensitive as an indirect indicator of infection and specific based on the results shown in Fig. 4. Furthermore, contact sentinels might be useful to study clinically inapparent colonization, as none of these mice became morbid at any time post infection despite colonisation of the lungs in numerous animals (Fig. 2).

This study shows for the first time that *R. pneumotropicus* infection results in a mean ratio of IgG2b to IgG1 above 1 in C57BL/6 but not in BALB/c mice indicating a Th1-prone immune response. In contrast, IgG2/IgG1 in BALB/c mice remained below 1 post infection suggesting a more balanced response. It is known already that BALB/c and C57BL/6 mice exhibit distinct genetically determined differences in their immune systems under physiological conditions, which includes higher amounts of interleukin (IL)-12 in C57BL/6 [30] and associated higher productions of IgG2c and 2b [31] inducing a Th1 immune response. However, based on the rapid progress of disease starting very early after infection, it is reasonable to hypothesize that other mechanisms but Th1 and Th2 immune responses were crucial for survival in the immunologically naïve mice of the described experimental infection. This is in agreement with the finding that the more Th1-prone C57BL/6 and the more Th2-prone BALB/c mice show comparable morbidities and pathologies.

We found high titres of *R. pneumotropicus*-specific IgA (above 150 Elisa) in the TNL of two surviving BALB/c mice 28 dpi in association with high numbers of this pathogen (above 10^4^ CFU/ml, results not shown). These results suggest maintenance of mucosal colonization of *R. pneumotropicus* despite high titers of specific IgA. As the specific bacterial load in the lung of the investigated survivors was significantly lower in C57BL/6 than in BALB/c mice, it is reasonable to hypothesize that the putative Th1-prone immune response in C57BL/6 mice is more efficient in restricting persisting lung infection.

## Conclusions

Many *R. pneumotropicus* strains recently isolated from laboratory mice in Germany carry the genes of the three known RTX toxins of this pathogen. Intranasal application of the *pnx*IA+ *pnx*IIA+ *pnx*IIIA+ *R. pneumotropicus* JF4Ni strain results in invasive and fatal infections in wt BALB/c and C57BL/6 mice associated with severe pneumonia and dissemination to extra-respiratory sites. In contrast to BALB/c mice, surviving C57BL/6 mice show a Th1-prone immune response and a bacterial load of the lung below 10^4^ CFU per g tissue after 28 dpi. The described model is ideal for future studies on virulence and protection elicited by vaccination.

## Methods

### Animals

Eight-week-old female wild type BALB/c mice obtained from Charles River laboratory (Sulzfeld Germany) and C57BL/6 mice from Janvier Labs (Le Genest-Saint-Isle France), both specific pathogen free, were caged randomly in 6 groups per strain including 2 uninfected and 4 infected groups. Each group contained 4 infection/placebo treated animals and one contact sentinel. Additionally, 10 week old female *P. pneumotropica-free* CD1 outbred mice (raised by the Fraunhofer Institute for Cell Therapy and Immunology, Leipzig, Germany) were included in this study as bedding sentinels (2 cages with 4 mice each). Each bedding-group was assigned to one mouse strain. The number of animals included in this study was based on the objectives to reveal putative differences between BALB/c and C57BL/6 mice in susceptibility, to evaluate sentinel monitoring and to obtain convalescence sera for a further immunoproteomics study. All animals were housed as described in a previous study in detail [32], which included ventilated cages with HEPA filters and air conditioning as well as ad libitum feeding and drinking. One week before infection blood samples were collected from the submandibular vein and stored at − 20 °C.

### Bacterial strains and culture media

*R. pneumotropicus* and *R. heylii were* cultivated as appropriate overnight at 37 °C on Columbia Blood Agar (COB) or in Brain Heart Infusion (BHI). Experimental infection was conducted with *R. pneumotropicus* strain JF4Ni grown in BHI until a concentration of 10^8^ colony forming units (CFU) per millilitre was reached. This strain was recently isolated from a German research facility with severe health problems associated with dyspnoea and increased mortality in P2X_2_/P2X_3_^Dbl−/−^ knockout but also wt mice [33]*.* Furthermore, *R. pneumotropicus* reference strain ATCC 35149 and 27 Biotype *Jawetz*- as well as 26 Biotype *Heyl* strains were included in this study (Additional file 3: Table S3).

### Infection

For experimental infection/mock treatment mice were anaesthetised by isoflurane inhalation. Mice were experimentally infected by intranasal application of 1 × 10^8^ CFU *R. pneumotropicus* JF4Ni in 25 μl phosphate buffered saline (PBS, 12.5 μl per nostril). Controls were inoculated with PBS only. Contact sentinels remained untreated, bedding sentinels were turned over to used bedding 7 days after inoculation. Experimentally infected and sentinel animals were sacrificed 4 and 8 weeks after experimental infection, respectively. For this, mice were anaesthesized through intraperitoneal application of 100 mg ketamin per kg body weight and 5 mg xylazin per kg body weight. They were bled by heart puncture and finally killed by cervical dislocation.

### Clinical examination and treatment

Every 12 h thorough adspection and weighing of mice was performed. Based on predefined criteria (Additional file 4: Table S4) clinical signs were scored. Mice with a cumulative clinical score of 3 or more were treated with flunixin meglumine for animal welfare reasons (5 mg per kg body weight every 12 h subcutaneously). The following end points led to euthanasia of respective animals: bleeding from orifices, paralysis, acute respiratory distress, cyanosis and 20% weight loss. Mice with a cumulative score above or equal 9 or a score above or equal 6 for 24 h were also killed for animal welfare reasons.

### Pathological examinations

For pathological studies brain, nasal conchae, lung, liver, spleen and kidney were collected, macroscopically examined and fixed in 4% CaCO_3_ buffered formalin. Tissues were embedded in paraffin, sectioned and stained with haematoxylin and eosin for light microscopy. Scoring of histopathological findings was conducted as specified in Additional file 5: Table S5.

### Cultural examinations

For semi quantitative cultural examinations, lung, brain, lymphonodi tracheobronchiales, liver, spleen, kidney and the genito-urinary tract of all BALB/c and C57BL/6 mice were collected and a fresh cut side was pressed on a COB plate, which was incubated for 24 h at 37 °C after streaking. For comparative analysis low, middle and high grades of detection of the typical colonies were scored with 1, 2 and 3 for each investigated tissue, respectively. The sum of the scores for all investigated tissues constitutes the total bacteriological score of each animal. Furthermore, quantitative cultural examinations were performed with the TNLs (300 μl PBS). For this, TNLs were serially diluted, spread on COB in duplicates and incubated for 24 h at 37 °C. Colonies were differentiated by MALDI-TOF-MS (Bruker microflex LT, Bremen).

### Antigen preparation and ELISA for detection of antigen-specific serum-IgG, serum-IgG2c and mucosal IgA

ELISAs were established for detection of serum IgG and mucosal IgA directed against *R. pneumotropicus* antigen. Either a whole cell extract or a concentrated culture supernatant of *R. pneumotropicus* JF4Ni was used as antigen.

For preparation of the antigens, 100 ml *R. pneumotropicus* culture were centrifuged at 2000 g for 15 min at 4 °C. The supernatant was concentrated 100 fold with a 30 kDa centrifugal filter unit (Amicon ultra), mixed with 0.5 ml 10 x protease inhibitor (Protease Inhibitor Cocktail with EDTA by Sigma Aldrich) per g cell pellet and dialyzed against 0.9% NaCl.

Whole cell extract was prepared from pelleted bacteria as described for *Streptobacillus moniliformis* previously [32]. Three hundred nanogramms of *P. pneumotropica* antigen (either whole cell extract or a concentrated culture supernatant) or casein (background measurement) were used per well to coat Corning Costar® assay plates in carbonate buffer (pH 8.1). Blocking with casein and washing of ELISA plates was conducted as described [32]. Twofold serial dilutions of sera and TNLs in PBS with 2 mM EDTA, 0.1% Tween20 and 0.1% bovine casein were applied to the ELISA plates including also reference sera/reference TNLs as well as negative controls. For detection of *P. pneumotropica*-specific serum-IgG, serum-IgG2c or TNL-IgA, plates were incubated for 1 h at RT with a 1:10,000 dilution of a HRP-conjugated goat anti-mouse IgG antibody (Jackson Immuno Research Laboratories), or a 1:10,000 dilution of a HRP-conjugated goat anti-mouse IgG2c antibody (Biorad; C57BL/6 mice only) or a 1:5000 dilution of HRP-conjugated goat anti-mouse IgA antibody (BIOMOL GmbH), respectively. ELISA plates were developed with 3,3′,5,5′-tetramethylbenzidine as described [32]. Absorbance was measured at 450 nm (reference at 630 nm).

The samples and the controls were measured in a duplicate series of four (seven for reference sera) twofold dilutions (starting with 1:200 for IgG and 1:50 for IgA). Sera collected from mice of a laboratory animal facility infected with the *R. pneumotropicus* strain JF4Ni were pooled and served as reference in the IgG and IgGc ELISA, TNL of an experimentally infected mouse from this experiment with a mean antibody titre as reference for the IgA ELISA. These reference samples were defined to include 100 ELISA units. Pooled sera and TNL samples from *R. pneumotropicus-*free mice were used as negative controls. Calculation of ELISA units for IgG and IgA was conducted as previously described [34].

### Determination of concentrations of IgG subtypes and respective ratios in sera of experimentally infected mice

Determination of IgG subtypes and calculation of respective ratios was conducted as described previously [32].

### Purification of DNA

The DNeasy Blood & Tissue Kit (Qiagen) was used according to the manufacturer’s instruction to purify DNA from *R. pneumotropicus and R. heylii* cultures.

### PCR screening for RTX genes *pnx*IA, *pnx*IIA and *pnx*IIIA

To detect the RTX toxin genes *pnx*IA, *pnx*IIA and *pnx*IIIA the primer pairs *pnx*IAF/*pnx*IAR, *pnx*IIAF/*pnx*IIAR and *pnx*IIIAF/*pnx*IIIARs (Additional file 6: Table S6) were used. The *pnx*IA and *pnx*IIA genes were amplified in full length from the DNA purified from different isolates (Additional file 3: Table S3), including strains genotyped in previous studies through 16S–23S rRNA internal transcribed spacer analysis [6, 35]. For detection of *pnx*IIIA amplification of an internal 1 kbp fragment was conducted. The *pnx* sequences were amplified by PCR with 30 cycles and Taq-polymerase (Invitrogen Thermo Scientific Fisher) as recommended by the manufacturer using the following conditions: denaturation at 95 °C for 30 s, annealing at 58 °C (*pnx*IA and *pnx*IIIA) or 61 °C (*pnx*IIA) for 30 s and elongation at 72 °C for 3:30 min (*pnx*IA), 6:30 min (*pnx*IIA) or 2 min (*pnx*IIIA).

### Statistical analysis

The Mann-Whitney test was performed to analyse differences between the different groups of mice. The Wilcoxon test was used for comparison of different time point values within the same group. The data in the Kaplan-Meyer survival and morbidity diagrams were analysed with the log rank test. Probabilities lower than 0.05 were considered significant, lower than 0.001 highly significant.

## Additional files

Additional file 1: **Table S1.** Scoring of semiquantitive bacteriological findings in *R. pneumotropicus* infected mice of the indicated strains succumbing to infection within the first week. (PDF 12 kb)

Additional file 2: **Table S2.** Scoring of semiquantitive bacteriological findings in *R. pneumotropicus* infected mice of the indicated strains surviving until the end of the observation period (4 weeks). (PDF 33 kb)

Additional file 3: **Table S3.** Scoring of clinical signs in mice infected with *R. pneumotropicus.* (PDF 10 kb)

Additional file 4: **Table S4** *R. pneumotropicus* and *R. heylii* strains used in this study. (PDF 10 kb)

Additional file 5: **Table S5** Scoring of catarrhal - purulent inflammations in mice infected with *R. pneumotropicus.* (PDF 4 kb)

Additional file 6: **Table S6:** Oligonucleotide primers used in this study. (PDF 16 kb)

## Abbreviations

BGPST: 5% BSA, 0.1% gelatine and 0.05% Tween20 in PBS
BHI: Brain heart infusion
CFU: Colony forming units
COB: Columbia Blood Agar
dpi: days post infection
ELISA: Enzyme-linked immunosorbent assay
FELASA: Federation of Laboratory Animal Science Association
IgG/A: Immune globuline G/A
IL: Interleukin
*P. multocida*: *Pasteurella multocida*
*P. pneumotropica*: *Pasteurella pneumotropica*
PBS: Phosphate buffered saline
PCR: Polymerase chain reaction
*R. pneumotropicus/heylii*: *Rodentibacter pneumotropicus/heylii*
RTX: Repeats in toxin
SD: Standard deviation
Th: T-helper cell
TNL: Tracheonasal lavage
