# Supplementary material for: Comparative analysis of humoral immune responses and pathologies of BALB/c and C57BL/6 wildtype mice experimentally infected with a highly virulent Rodentibacter pneumotropicus (Pasteurella pneumotropica) strain
Source: BMC Microbiol. 2018 May 30;18:45. doi: 10.1186/s12866-018-1186-8 (PMC5977748; doi:10.1186/s12866-018-1186-8)
Supplement: Supplementary file 1 — Table S1. Scoring of semiquantitive bacteriological findings in R. pneumotropicus infected mice of the indicated strains succumbing to infection within the first week. (PDF 12 kb) [file 12866_2018_1186_MOESM1_ESM.pdf]

Table S1 Scoring of semiquantitive bacteriological findings in *R. pneumotropicus* infected mice of the indicated strains succumbing to infection within the first week

strain	No.	brain	lung	lymph node	liver	spleen	kidney	genito-urinary tract	total score
BALB/c	714	3	3	3	3	0	0	0	12
	719	2	3	3	2	0	1	0	11
	720	1	3	3	3	0	0	0	10
	721	2	2	3	1	0	0	2	10
	722	0	2	3	2	2	1	3	13
	725	1	0	3	2	0	0	0	6
	727	2	3	3	2	0	0	3	13
	729	2	2	3	2	0	1	2	12
	731	3	3	3	3	2	0	3	17
C57BL/6	1748	1	1	2	0	0	0	1	5
	1753	1	2	1	0	0	0	1	5
	1754	2	2	2	0	0	0	0	6
	1755	2	2	1	0	0	0	0	5
	1756	0	3	2	1	0	1	0	7
	1758	1	1	0	0	0	0	2	4
	1761	1	2	3	1	0	0	0	7
	1764	1	2	1	2	1	2	1	10

Early losses of BALB/c and C57BL/6 mice had mean total scores of 11.6 (SD 3.0) and 6.1 (SD 1.9), respectively, and these differences were significant ($p = 0.0027$). Low, middle and high grades of detection of typical colonies received scores of 1, 2 and 3, respectively.
