# Supplementary material for: Comparative analysis of humoral immune responses and pathologies of BALB/c and C57BL/6 wildtype mice experimentally infected with a highly virulent Rodentibacter pneumotropicus (Pasteurella pneumotropica) strain
Source: BMC Microbiol. 2018 May 30;18:45. doi: 10.1186/s12866-018-1186-8 (PMC5977748; doi:10.1186/s12866-018-1186-8)
Supplement: Supplementary file 2 — Table S2. Scoring of semiquantitive bacteriological findings in R. pneumotropicus infected mice of the indicated strains surviving until the end of the observation period (4 weeks). (PDF 33 kb) [file 12866_2018_1186_MOESM2_ESM.pdf]

Table S2 Scoring of semiquantitive bacteriological findings in *R. pneumotropicus* infected mice of the indicated strains surviving until the end of the observation period (4 weeks)

strain	No.	brain	lung	lymph node	liver	spleen	kidney	genito-urinary tract	total score
BALB/c	715	1	1	0	1	0	2	3	8
	716	0	2	1	1	0	0	0	4
	717	1	2	3	0	0	0	0	6
	724	0	2	2	0	0	0	0	4
	726	0	2	0	0	0	0	0	2
	730	0	3	3	0	0	0	0	6
	732	0	2	2	2	0	1	3	10
C57BL/6	1749	1	1	0	2	0	3	3	10
	1750	0	0	0	0	0	0	0	0
	1751	0	1	0	0	0	0	3	4
	1759	0	1	1	0	0	0	0	2
	1760	0	1	0	0	0	0	1	2
	1763	0	0	1	0	0	0	0	1
	1765	0	0	0	0	0	0	0	0
	1766	0	0	0	0	0	0	0	0

Surviving BALB/c and C57BL/6 had mean total scores of 5.7 (SD 2.7) and 2.4 (SD 3.4), respectively, and these differences were significant (p = 0.036). Low, middle and high grades of detection of typical colonies received scores of 1, 2 and 3, respectively.
