# Supplementary material for: Comparative analysis of humoral immune responses and pathologies of BALB/c and C57BL/6 wildtype mice experimentally infected with a highly virulent Rodentibacter pneumotropicus (Pasteurella pneumotropica) strain
Source: BMC Microbiol. 2018 May 30;18:45. doi: 10.1186/s12866-018-1186-8 (PMC5977748; doi:10.1186/s12866-018-1186-8)
Supplement: Supplementary file 3 — Table S3. Scoring of clinical signs in mice infected with R. pneumotropicus. (PDF 10 kb) [file 12866_2018_1186_MOESM3_ESM.pdf]

Table S3 *R. pneumotropicus* and *R. heylii* strains used in this study

Species	Strains	Reference
R. pneumotropicus	ATCC 35149	DSMZ
	JF4Ni/15	Institute for Bacteriology and Mycology, Faculty of Veterinary Medicine, Leipzig
	JF10GVG/16, JF17GVG/16, JF18GVG/16, JF21GVG/17, JF22GVG/17, JF23GVG/17, JF24GVG/17, JF25GVG/17	GVG Diagnostics GmbH
	1596/07, 217/08, 607/10, 567/11, 665/11, 691/11, 695/11, 847/11, 1009/11, 1012/11, 1023/11, 394/12, 1526/12, 1528/12, 1550/12, 530/13	Benga *et al.* (2012) [34], Benga *et al*. (2013) [6]
R. heylii	JF1Fe/16, JF19GVG/16, JF20GVG/16, JF26GVG/17, JF27GVG/17	GVG Diagnostics GmbH
	218/08, 314/08, 520/08, 1825/08, 450/10, 256/11, 490/11, 543/11, 566/11, 568/11, 622/11, 666/11, 693/11, 696/11, 705/11, 1070/11, 1527/12, 1528/12, 1552/12, 526/13	Benga *et al.* (2012) [35], Benga *et al.* (2013) [6]
