# Supplementary material for: Comparative analysis of humoral immune responses and pathologies of BALB/c and C57BL/6 wildtype mice experimentally infected with a highly virulent Rodentibacter pneumotropicus (Pasteurella pneumotropica) strain
Source: BMC Microbiol. 2018 May 30;18:45. doi: 10.1186/s12866-018-1186-8 (PMC5977748; doi:10.1186/s12866-018-1186-8)
Supplement: Supplementary file 4 — Table S4 R. pneumotropicus and R. heylii strains used in this study. (PDF 10 kb) [file 12866_2018_1186_MOESM4_ESM.pdf]

Table S4 Scoring of clinical signs in mice infected with *R. pneumotropicus*

**Parameters**	**0**	**1**	**2**
body weight	steady or increase	≥5% decrease	≥20% decrease
coat	smooth, fitting, shining	rough, decreased grooming	ruffled, no grooming
breathing	normal, rhythmic	fast, superficial	fast, abdominal
dehydration	normal skin elasticity	degraded skin elasticity	persisting skin fold, caved in eyes
posture	normal	bended back	cowering
eyes	normal	tweak, discharge	caved in
behaviour	normal, active, curious	depression	apathy, isolation from group
motion	no anomaly	incoordination	apraxia, tumbling
