# Supplementary material for: Comparative analysis of humoral immune responses and pathologies of BALB/c and C57BL/6 wildtype mice experimentally infected with a highly virulent Rodentibacter pneumotropicus (Pasteurella pneumotropica) strain
Source: BMC Microbiol. 2018 May 30;18:45. doi: 10.1186/s12866-018-1186-8 (PMC5977748; doi:10.1186/s12866-018-1186-8)
Supplement: Supplementary file 5 — Table S5 Scoring of catarrhal - purulent inflammations in mice infected with R. pneumotropicus. (PDF 4 kb) [file 12866_2018_1186_MOESM5_ESM.pdf]

Table S5 Scoring of catarrhal - purulent inflammations in mice infected with *R. pneumotropicus*

	**Score**
mild, focal	1
mild, multifocal or diffuse	2
mild to moderate, focal	2
mild to moderate, multifocal or diffuse	3
moderate, focal	3
moderate, multifocal or diffuse	4
mild, focal with necrosis	4
moderate to severe, multifocal or diffuse	5
moderate to severe, multifocal or diffuse with necrosis	5
severe, multifocal or diffuse	6
severe, focal with necrosis	6
moderate to severe, multifocal or diffuse with necrosis	6
severe, multifocal or diffuse with necrosis	7
