# Supplementary material for: Comparative analysis of humoral immune responses and pathologies of BALB/c and C57BL/6 wildtype mice experimentally infected with a highly virulent Rodentibacter pneumotropicus (Pasteurella pneumotropica) strain
Source: BMC Microbiol. 2018 May 30;18:45. doi: 10.1186/s12866-018-1186-8 (PMC5977748; doi:10.1186/s12866-018-1186-8)
Supplement: Supplementary file 6 — Table S6: Oligonucleotide primers used in this study. (PDF 16 kb) [file 12866_2018_1186_MOESM6_ESM.pdf]

Table S6 Oligonucleotide primers used in this study

Primer	Oligonucleotide sequence (5´-3´)	Target
*pnx*IAF	TATTAACCCGGGACTGAATATTTAGATGAAAT	*pnx*IA
*pnx*IAR	TATTAACTCGAGTAATATAGATTGATAATTAG	*pnx*IA
*pnx*IIAF	TAATATCCCGGGACTTTTCTCCATATTATTAA	*pnx*IIA
*pnx*IIAR	TATGTGCTCGAGAATAATCTCATATTGGCTTT	*pnx*IIA
*pnx*IIIAF	CAGGATGCGGCCGCTTCGACTACATTAAAAGTAT	*pnx*IIIA
*pnx*IIIARs	CTGTTACTGTATCACCTGCTTTG	*pnx*IIIA
